# 1767. Ventilation in Non-Healthcare Congregate Settings: Capacity-Building Assessments to Reduce COVID-19 Exposure Risks, California 2022 – 2023

**DOI:** 10.1093/ofid/ofad500.1598

**Published:** 2023-11-27

**Authors:** Jared G Wiegand, Kyle T Peerless, Robert Stepp, Elon Ullman, Taria Greenberg, Cameron R Stainken, Christina Armatas, Kristin J Cummings, Wendy M Chung

**Affiliations:** California Department of Public Health, Richmond, California; California Department of Public Health, Richmond, California; California Department of Public Health, Richmond, California; California Department of Public Health, Richmond, California; California Department of Public Health, Richmond, California; California Department of Public Health, Richmond, California; California Department of Public Health, Richmond, California; California Department of Public Health, Richmond, California; California Department of Public Health, Richmond, California

## Abstract

**Background:**

Optimizing indoor air quality has been increasingly recognized as a cornerstone strategy to minimize aerosol transmission of SARS-CoV-2, particularly in non-healthcare congregate settings. Expertise often is lacking within local health departments (LHDs), to provide ventilation guidance to such facilities.

**Methods:**

We offered non-regulatory ventilation assessments by state industrial hygienists (IHs) to 58 LHDs. Interested LHDs selected 1-2 non-healthcare congregate facilities, prioritized by: administrator interest, whether they were publicly funded, or had past COVID-19 outbreaks. We requested on-site participation of LHD and facility staff. State IHs presented educational material at pre-visit meetings and during the visits. Findings were collected systematically, and recommendations shared in reports and post-visit debriefings. Participants completed post-visit surveys.

**Results:**

We visited 25 facilities in 19 counties, including 8 homeless shelters, 7 jails, 8 schools, 1 campground, and 1 behavioral health facility. Most (68%) were not operating heating, ventilation, and air conditioning (HVAC) systems continuously and air dampers were closed or obstructed in 4 (16%) facilities. Half of facilities had insufficient HVAC maintenance. Only 7 (28%) facilities used Minimum Efficiency Reporting Value (MERV) 13 filters. Of residential facilities, 3 (19%) had medical isolation spaces equipped with dedicated air handlers. Of non-correctional facilities, 11 (61%) used portable air cleaners. No facilities had implemented staff training on ventilation measures. In post-visit surveys, LHDs reported gaining a better understanding of actions that facilities can take to improve ventilation practices, which could also be generalized to other settings.

**Conclusion:**

State IHs can have an important impact in bolstering public health workforce knowledge of principles of ventilation assessments. Common inadequacies in ventilation practices may occur in a variety of non-healthcare congregate settings, increasing the risk of SARS-CoV-2 transmission and outbreaks in vulnerable persons. Demonstrative site visits can effectively provide practical training to LHDs and inform the development of tools to build public health capacity to guide under-resourced settings.

**Disclosures:**

**All Authors**: No reported disclosures

